# In vitro activities of *Traganum nudatum* and *Mentha pulegium* extracts combined with amphotericin B against *Candida albicans* in production of hydrolytic enzymes

**DOI:** 10.18502/cmm.6.3.4499

**Published:** 2020-09

**Authors:** Ikram Tefiani, Sidi Mohammed Lahbib Seddiki, Moustafa Yassine Mahdad

**Affiliations:** 1 Department of Antifungal Antibiotic, Physico-Chemical Synthesis, and Biological Activity, University of Tlemcen, Tlemcen, Algeria; 2 University Center of Naâma, Naâma, Algeria; 3 Department of Physiology, Physiopathology, and Biochemistry of Nutrition, University of Tlemcen, Tlemcen, Algeria

**Keywords:** Amphotericin B, *Candida albicans*, Hydrolytic enzymes, *Mentha pulegium*, *Traganum nudatum*

## Abstract

**Background and Purpose:**

*Candida albicans* is an important microorganism in the normal flora of a healthy subject; however, it has an expedient pathogenic character that induces hydrolytic virulence. Regarding this, the present study aimed to find an in vitro alternative that could reduce the virulence of this yeast.

**Materials and Methods:**

For the purpose of the study, the effect of amphotericin B (AmB) combined with the extract of *Traganum nudatum* (E1) or *Mentha pulegium* (E2) was evaluated against the hydrolytic activities of esterase, protease, and phospholipase. This effect was determined by calculating the minimum inhibitory concentration (MIC), used to adjust the extract/AmB mixtures in culture media.

**Results:**

The evaluated Pz values, which corresponded to the different enzymatic activities, showed a decrease in the hydrolytic activities of *C. albicans* strains after the addition of E1/AmB and E2/AmB combinations at descending concentrations (lower than the obtained MICs).

**Conclusion:**

Based on the findings, it would be possible to reduce the pathogenesis of this species without destabilizing the balance of the flora.

## Introduction

*Candida albicans* is the most isolated opportunistic fungal pathogen in humans. This species is a harmless commensal organism in the gastrointestinal and genitourinary tracts; accordingly, it is a mandatory commensal species [ 1 , 2 ]. In a normal case, there is a fair balance between fungal virulence and host defense mechanisms [ 3 ]. However, in case of the disturbance of the host environment or immune dysfunction, the fungal burden will be higher than the commensal levels. Under such conditions, *C. albicans* can proliferate and invade deep sites and induce a high mortality rate [ 4 , 5 ].

Several virulence factors, particularly the hydrolytic activity of esterases, aspartyl proteases, and phospholipases, account for the pathogenesis of this species [ 6 ]. These enzymes cause the hydrolysis of ester bonds in glycerophospholipids and acylglycerols [ 7 ], leading to the breakage of the membranes of the epithelial cells, and consequently *C. albicans* penetration into the cytoplasm [ 8 ]. In addition, proteases cause the destruction of the host immune tissue [ 9 ]. On the other hand, invasive fungal infections are difficult to diagnose and challenging to treat [ 10 ]. Although amphotericin B (AmB) remains the first-line antifungal treatment, its applicability is associated with highly toxic side effects [ 11 ].

*Candida albicans* plays an essential role in the microbial flora of a healthy person. The achievement of knowledge regarding hydrolytic enzymes would help develop antifungal agents to inhibit these virulence factors [ 12 ]. Regarding this, it is imperative to look for new alternatives to develop more effective therapeutic strategies that are less aggressive for the body [ 13 ]. Accordingly, the present study aimed to preserve the viability of *C. albicans* while inhibiting the virulent potential of hydrolytic enzymes. To this end, the extracts of two selected plants, namely *Mentha pulegium* and *Traganum nudatum*, with confirmed antifungal properties [ 14 , 15 ], were tested in combination with AmB at concentrations below the minimum inhibitory concentrations (MIC).

## Materials and Methods

**Plant extracts**

This study involved the use of two plant extracts, including the ethyl acetate extract of *Traganum nudatum* (E1) and the hydromethanolic extract of *Mentha pulegium* (E2). The E1 and E2 were chosen due to having confirmed antifungal activities against *C. albicans* [ 14 , 15 ]. The stock solutions of the extracts were prepared by dissolving E1 in dimethylsulfoxide (DMSO, Sigma-Aldrich) and E2 in distilled water. Subsequently, they were sterilized by filtration on a filter with a porosity of 0.20 µm.

**Inocula preparation**

Four strains of *C. albicans* (i.e., A6, A8, A11, and A13) were investigated in this study. In addition, the reference strain of *C. albicans* ATCC 10231 was utilized as a positive control for the various tests. Two fungal suspensions were prepared from 18-hour-old yeast cells. The first suspension was prepared at the concentration of 10^6^ cells/mL for the esterase and protease tests [ 16 , 17 ], while the second was made at 10^8^ cells/mL for the phospholipase test [ 18 ].

**Determination of minimum inhibitory concentration of extracts**

The MIC, defined as the lowest concentration of the extract capable of interrupting any visible growth of microorganisms, was evaluated using the broth dilution antifungal susceptibility testing, following the protocol M27-A3 of the Clinical Laboratory Standard Institute (2008) [ 19 ] with some modifications. This protocol was adapted to determine the MICs of the E1 and E2 extracts against the *C. albicans* strains. Briefly, sterile 96-well microplates and Roswell Park Memorial Institute 1640 medium (RPMI 1640, Sigma-Aldrich), buffered to a pH of 7 with morpholine propane sulfonic acid (MOPS, Sigma-Aldrich), were used.

For the purpose of the study, 100 μl of RPMI 1640 was placed in the microplate wells. In the next stage, 100 μL of the antifungal solution or the extract to be tested was added to the 1^st^ wells of the microplates. These were followed by a series of dilutions of ½ from well to well so that the final concentrations of the extracts in the wells were between 3 and 0.02 mg.mL^-1^. The last lines of the microplates served as positive and negative controls, respectively. The microplates were sealed and then incubated at 37°C for 24 h. The MIC evaluation was carried out with the naked eye.

**Minimum inhibitory concentration of extracts in association with Amphotericin B**

The stock solution of AmB was prepared by dissolving it in DMSO. The MIC of the AmB used for the following tests (0.5 µg.mL^-1^) was that of the reference strain, namely *C. albicans* ATCC10231 [ 20 ]. The use of the extracts at decreasing MICs and the maintenance of this value for AmB, and vice versa, made it possible to have the best combinations to achieve the objective.

The experiment was carried out in two stages. In the first stage, based on the MICs calculated previously (previous section), the extracts were prepared at decreasing concentrations while maintaining the same final concentration for AmB (0.5 µg.mL^-1^). In the second stage, this procedure was reversed; in other words, the concentrations of the extracts were maintained at the same level, while AmB was prepared at decreasing concentrations (0.5-0.003 µg.mL^-1^). Briefly, 100 μL of RPMI 1640 medium was deposited in the wells of the microplates; then, 100 μL of the extract, either E1 or E2, was added to the first wells and a series of dilution of ½ was carried out. In the next stage, 100 μL AmB and 100 μL inoculum were added to the wells. Finally, positive and negative control tests were included. The microplates were then sealed and incubated at 37°C for 24 h.

**Hydrolytic activities**

The direct agar contact technique [ 21 ] was adopted to assess the effects of E1/AmB and E2/AmB mixtures on the activity of hydrolytic enzymes of *C. albicans* strains. Tween 80 opacity test and the agar, containing bovine serum albumin, were respectively used for the evaluation of the activities of esterase and protease [ 16 , 17 ]. Furthermore, the evaluation of the phospholipase activity was accomplished using Sabouraud dextrose agar, supplemented with egg yolk [ 18 ].

For each enzymatic activity, two tests were carried out in parallel. The first test involved the evaluation of the enzymatic activity of the strains in the absence of E1, E2, or AmB. On the other hand, the second test consisted of the separation examination of the enzymatic activity of the strains, in the presence of the E1/AmB and E2/AmB mixtures. To this end, 1 mL of the extract solution and 1 mL of AmB were added to the agar prior to each test at concentrations corresponding to the MICs previously evaluated; all tests were performed in duplicate. The activity levels of the hydrolytic enzymes were established according to the Pz index, which was calculated by the ratio of the diameter of the colony to the diameter of the colony plus that of the precipitation zone [ 22 ].

**Statistical analysis**

GEN STAT Discovery Edition 3 statistical software was used for data analysis. The results were presented as mean±standard error of mean. The comparison between the means was performed using the ANOVA and Duncan's multiple range test. The significance level was set at a *p-value* of < 0.01.

## Results

**Determination of minimum inhibitory concentration of extracts**

The concentration of 0.5 µg.mL^-1^ was taken as the MIC of AmB. The results obtained showed that both extracts had inhibitory effects against all the tested strains of *C. albicans*. According to these results, the MIC for E1 was 1.5 mg.mL^-1^. However, that of E2 was twice as high (3 mg.mL^-1^). These results were observed in all strains of *C. albicans*, including the reference one.

**Minimum inhibitory concentration of extracts in association with amphotericin B**

In this part, the experiment was carried out using the extracts at decreasing concentrations while maintaining the same final concentration of AmB (0.5 µg.mL^-1^) and vice versa. The MICs obtained for the AmB/E1 and E1/AmB mixtures were 0.5/0.002 and 0.375/0.25 mg.µg^-1^.mL^-1^, respectively (Table 1). Furthermore, the MIC of the AmB/E2 mixture was obtained as 0.5/0.75 µg.mg^-1^.mL^-1^, whereas that of E2/AmB was estimated at 1.5/0.25 mg.µg^-1^.mL^-1^ with respect to all the strains used.

**Hydrolytic activities**

**Figure 1:**
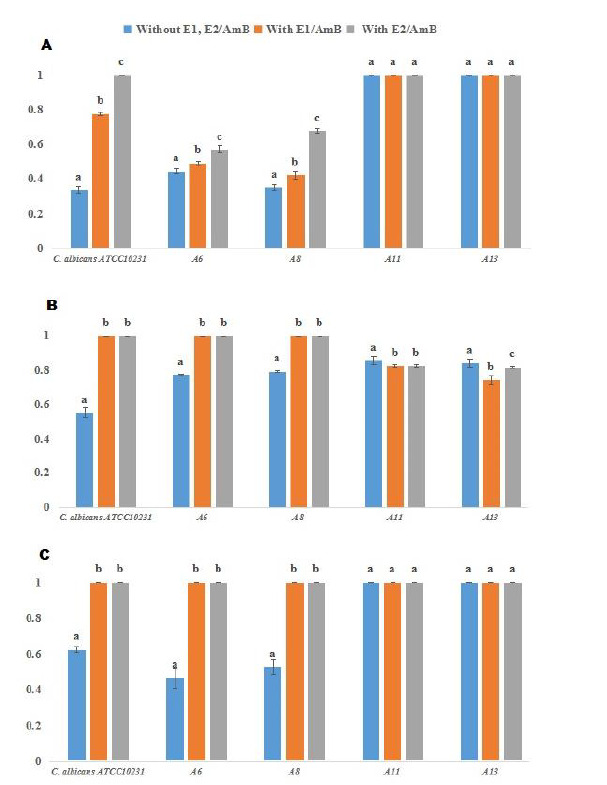
In vitro assessment of the hydrolytic activities of *C. albicans* in the absence and presence of *Mentha pulegium*/amphoceritin B and *Traganum nudatum*/amphoceritin B mixtures; A) esterase activity, B) protease activity, and C) phospholipase activity (Vertical bars show standard errors. Histogram bars of the same color with the same letters do not differ significantly from Duncan's Multiple Range Test at 1% probability level. The x-axis represents the strains of *C. albicans* used.)

The hydrolytic activities of esterase, protease, and phospholipase are respectively presented in figures 1, 2, and 3. The protease hydrolytic activity was noted in all the strains; however, two strains (i.e., A11 and A13) did not show any activity with respect to esterase and phospholipase. For the esterase activity, three strains (i.e., A6, A8, and *C. albicans* ATCC10231) exhibited a strong esterase activity (Pz≤0.63). The Pz values varied between 0.33±0.02 and 0.44±0.01. However, this activity was negative in A11 and A13 (Pz=1). Besides, all the studied strains showed positive protease activity. Strong activity was recorded in *C. albicans* ATCC10231 (Pz=0.55±0.02).

Strains A6 and A8, on the other hand, had average activities with the respective Pz values of 0.77±0.004 and 0.79±0.007, respectively. However, a slight activity was observed in A11 and A13 (Pz values of 0.85±0.02 and 0.84±0.02, respectively). In addition, phospholipase activity was considered to be strong in *C. albicans* ATCC10231, A6 and A8 with the respective Pz values of 0.62±0.01, 0.46±0.05, and 0.52±0.04. In contrast, A11 and A13 showed no phospholipase activity (Pz=1).

**Effects of amphotericin B/extract mixtures on enzyme activity**

It is important to note that the effect of AmB/extract combination led to interesting results. In this regard, the use of a fixed concentration for extracts and reduction of AmB concentration did not seem to have an anti-virulent effect against the hydrolytic activity of *C. albicans*. Therefore, only the results of the reverse combinations, namely E1/AmB and E2/AmB, at the respective concentrations of 0.375/0.25 and 1.5/0.25 mg.µg^-1^.mL^-1^ were discussed. In addition, this seems more important because the toxicity of AmB at 0.25 µg.mL^-1^ is minimal, compared to that at 0.5 µg.mL^-1^ [ 23 ].

The results obtained in this experiment showed that the addition of 0.375/0.25 mg.µg^-1^.mL^-1^ of E1/AmB mixture in the culture medium significantly contributed to the reduction of the esterase activity of *C. albicans* ATCC10231, which was changed from strong to medium (Pz=0.77±0.01). For strains A6 and A8, the non-significant increase (P<0.01) in Pz values did not modify the level of hydrolytic activity. In addition, no esterase activity was observed in strains A11 and A13. On the other hand, this activity was completely inhibited in *C. albicans* ATCC10231 when E2/AmB mixture was introduced into the culture medium at a concentration of 1.5/0.25 mg.µg^-1^.mL^-1^. For A6, a slight increase was observed in Pz value. This value was doubled (Pz=0.67±0.07) for the A8 strain. However, these results remained significant (P<0.01) and seemed to have a reducing effect on the hydrolytic activity of esterase (Figure 1A). For A11 and A13, no change was observed in esterase activity, which was zero.

The addition of E1/AmB and E2/AmB mixtures to the culture medium at the respective concentrations of 0.375/0.25 and 1.5/0.25 mg.µg^-1^.mL^-1^ induced a complete inhibition of protease activity in *C. albicans* ATCC10231, A6, and A8 strains (Pz=1). In contrast, A11 and A13 retained a slight level of protease activity (Figure 1B). In addition, E1/AmB mixture significantly (P<0.01) induced an increase in this activity in A13. According to Figure 1B, the protease activity was modified from one strain to another depending on the addition of E1/AmB and E2/AmB mixtures to the culture medium. On the other hand, the results obtained with regard to the phospholipase activity (Figure 1C) revealed its inhibition under the effect of E1/AmB and E2/AmB mixtures at the respective concentrations of 0.375/0.25 and 1.5/0.25 mg.µg^-1^.mL^-1^.

The influence of E1/AmB and E2/AmB mixtures on phospholipase activity was remarkable. This activity was completely inhibited in *C. albicans* ATCC10231, A6, and A8 (Pz=1). The very significant increase in Pz values (P<0.01) suggests that these mixtures may have an interesting anti-virulent effect against the hydrolytic activity of *C. albicans*. Otherwise, A11 and A13 did not show any activity, either in the presence of the mixtures or in their absence.

**Table 1:** Minimum inhibitory concentrations of E1 and E2 extracts in association with amphotericin B

Combination *Extract & AmB*	AmB/E1 (µg.mg^-1^.mL^-1^)	E1/AmB (mg.µg^-1^.mL^-1^)	AmB/E2 (µg.mg^-1^.mL^-1^)	E2/AmB (mg.µg^-1^.mL^-1^)
MIC	0.5/0.002	0.375/0.25	0.5/0.75	1.5/0.25

## Discussion

Recently, Mouderas et al. [ 14 ] showed that *C. albicans* ATCC10231 was resistant to 0.5 mg.mL^-1^ of *Traganum nudatum* ethyl acetate extract. However, unlike the results of this study, the methanolic extract of *Mentha pulegium* did not show any antimicrobial effect against *C. albicans* at a concentration of < 50 mg.mL^-1^ [ 24 ]. From a practical point of view, it is important to note that antibiotics are sometimes used in combinations because of their additive or synergistic effects [ 25 ]. The MICs of extracts in association with AmB revealed variable inhibitory effects among the different combinations. Guo et al. [ 26 ] reported a synergistic effect for Thymol/AmB combination evaluated by the microdilution method against the clinical isolates of *C. albicans*. Additionally, other researchers have found that the association of AmB with the essential oil of Melaleuca alternifolia had a fungicidal additive effect. This synergy is considered to be a real gain for the therapy of *C. albicans* infections [ 27 ].

*Candida albicans* exists commensally in many healthy humans; however, there is no doubt that the secretion of hydrolytic enzymes is one of the virulence factors in this yeast [ 28 , 29 ]. The obtained results revealed the hydrolytic activity of proteases in all strains; nonetheless, two strains (i.e., A11 and A13) showed no activity towards esterase and phospholipase. Recently, Pandey et al. (2018) reported that 56.25% of the strains were strong producers of esterase [ 30 ]. For protease activity, its expression was found in the majority of the clinical isolates [ 31 ]. On the other hand, phospholipase, which is the enzyme responsible for phospholipid hydrolysis by *C. albicans*, was detected in all isolates of this species [ 32 , 33 ]. However, Price et al. in 1982 reported phospholipase activity in only 30% of the investigated isolates [ 22 ].

For Cui et al. [ 34 ], synergistic combinations based on experimental methods were a formidable challenge in terms of cost and time. This study showed that the addition of E1/AmB and E2/AmB mixtures to the culture medium at the respective concentrations of 0.375/0.25 1.5/0.25 mg.µg^-1^.mL^-1^ induced a partial or total reduction in hydrolytic activities. This suggests that the anti-virulent effect of these associations depends on the strains and enzymes. The decrease in the enzymatic activity of *C. albicans* isolates could considerably reduce their pathogenic power [ 35 ]. In parallel, secondary metabolites may be involved in the inhibition of the pathogen's enzymes [ 36 ]. In 2008, Mohan and Ballal [ 37 ] revealed a strong reduction in the synthesis of phospholipases and proteases in *C. albicans* after adding the raw extract of Eugenia uniflora to the culture media. Other research has shown that the enzymatic activity in this species, treated with essential oils, was significantly reduced [ 38 ].

## Conclusion

Hydrolytic enzymes, in particular esterases, proteases, and phospholipases, are biochemical factors in the virulence of *Candida albicans*. Since this species is part of the microbial flora of a healthy person, an immune imbalance increases the risk of opportunistic infection. Therefore, it is better to keep this yeast in the microbial flora while reducing its hydrolytic virulence, rather than eradicating it totally. Synergistic combinations with anti-virulent effect of antifungal agent and plant extracts against *C. albicans* can have a positive role in reducing its pathogenesis. As our results demonstrated, the E1/AmB and E2/AmB combinations with the respective concentrations of 0.375/0.25 and 1.5/0.25 mg.µg^-1^.mL^-1^ presented a remarkable in vitro effect against the hydrolytic activities of *C. albicans*, thereby reducing the pathogenesis of this species and not destabilizing the flora equilibrium.
